# Childhood Cognition and Age-Related Change in Standing Balance Performance From Mid to Later Life: Findings From a British Birth Cohort

**DOI:** 10.1093/gerona/gly275

**Published:** 2018-12-08

**Authors:** Joanna M Blodgett, Diana Kuh, Rebecca Hardy, Daniel H J Davis, Rachel Cooper

**Affiliations:** MRC Unit for Lifelong Health and Ageing at UCL, London, UK

**Keywords:** Life course, Balance, Cognition, Age-related decline

## Abstract

**Background:**

Cognitive processing plays a crucial role in the integration of sensory input and motor output that facilitates balance. However, whether balance ability in adulthood is influenced by cognitive pathways established in childhood is unclear, especially as no study has examined if these relationships change with age. We aimed to investigate associations between childhood cognition and age-related change in standing balance between mid and later life.

**Methods:**

Data on 2,380 participants from the MRC National Survey of Health and Development were included in analyses. Repeated measures multilevel models estimated the association between childhood cognition, assessed at age 15, and log-transformed balance time, assessed at ages 53, 60–64, and 69 using the one-legged stand with eyes closed. Adjustments were made for sex, death, attrition, anthropometric measures, health conditions, health behaviors, education, other indicators of socioeconomic position (SEP), and adult verbal memory.

**Results:**

In a sex-adjusted model, 1 standard deviation increase in childhood cognition was associated with a 13% (95% confidence interval: 10, 16; *p* < .001) increase in balance time at age 53, and this association got smaller with age (cognition × age interaction: *p* < .001). Adjustments for education, adult verbal memory, and SEP largely explained these associations.

**Conclusions:**

Higher childhood cognition was associated with better balance performance in midlife, with diminishing associations with increasing age. The impact of adjustment for education, cognition and other indicators of SEP suggested a common pathway through which cognition is associated with balance across life. Further research is needed to understand underlying mechanisms, which may have important implications for falls risk and maintenance of physical capability.

The ability to balance underlies nearly all physical movement at each stage of life from an infant learning to stand to an older adult trying to avoid a fall. Age-related decline in balance ability is a particular concern due to high incidence of falls in older individuals (1), and growing evidence that poor balance performance is also associated with higher disability, morbidity, and mortality rates (2,3). Yet balance remains an under-recognized and frequently overlooked aspect of physical health (4,5).

Neural processing plays a crucial role in integrating the sensory input and motor output involved in successfully balancing (6). Given that the brain undergoes significant development throughout childhood and adolescence (7), the neural processes involved in balance could be a result of early establishment of synapses and neural connectivity (8). Further investigation of neurological development and its contribution to balance could identify a period in early life, during which cognitive processes involved in balance may be formed.

Previous analyses of the MRC National Survey of Health and Development (NSHD) (9,10) and a Danish cohort study (11) have demonstrated that higher cognition in early life (at ages 8–18) was associated with better balance performance at ages 53 and 50, respectively. However, no study has investigated these associations with balance in later life nor investigated serial measures of balance ability to see if these associations change with age. Studies examining age-related decline in physical capability have prioritized more proximal age-related factors, such as chronic health conditions, health care utilization, and other psychosocial factors (12–14). Conversely, the life course approach suggests that physical and social factors in early and midlife contribute to health and disease risk in later life (15). Here, childhood cognition may be associated with peak balance ability and subsequent age-related change for several reasons: childhood cognition is considered to be an initial indicator of lifetime cognitive ability, it is associated with lifelong socioeconomic pathways and it is predictive of health behaviors, all of which relate to balance ability (16–18).

The objectives of this analysis were to (a) examine the associations between childhood cognition and standing balance at three ages in mid and later life; (b) assess whether these associations change with age; and (c) investigate the impact of adjusting for adult cognition, education, anthropometric measures, and sociobehavioural factors.

## Methods

### Study Sample

The NSHD is a nationally representative study of 5,362 males and females born within 1 week in March 1946 in England, Scotland, and Wales. Study members have been assessed up to 24 times since birth and were most recently examined by trained research nurses at ages 53 (*n* = 2,988), 60–64 (*n* = 2,229) and 69 (*n* = 2,149). Reasons for nonparticipation include death, emigration, refusal, and incapacity [(19) Supplementary Figure 1]. Participants provided written consent at each data collection. Ethical approval for the most recent visit (at age 69) was given by Queen Square Research Ethics Committee (13/LO/1073) and Scotland A Research Ethics Committee (14/SS/1009).

### Measurement of Balance

At ages 53, 60–64, and 69, participants were asked to fold their arms across their chest and, when indicated by the nurse, stand on their preferred leg and raise the contralateral foot off the floor for a maximum of 30 seconds. The test was undertaken first with eyes open and then repeated with eyes closed, with the eyes closed score used in these analyses due to a ceiling effect with eyes open. Balance time was measured in seconds at age 53 and in milliseconds at ages 60–64 and 69. At age 53, 93.4% of participants who underwent assessment completed an eyes closed balance test, 94.8% completed the test at age 60–64, while 92.3% completed the test at age 69. Reasons for noncompletion of tests were recorded by the nurse. Those who were unable to complete the test due to health reasons were excluded from primary analyses.

### Childhood Cognition

Childhood cognition was ascertained at age 15 when participants completed the Alice Heim (AH4) test of fluid intelligence, the Watts–Vernon reading test, and a study-specific test of mathematical ability (17,20). Each test score was standardized and then summed to create an overall cognitive score which was then standardized to the analytical sample (mean of 0, *SD* [standard deviation] of 1). Consistent with other NSHD analyses, if the score was missing at age 15, scores from comparable examinations of global cognition at ages 11 or 8 (*n* = 165) were used (17,21,22).

### Covariates

Covariates were chosen based on a review of the literature and previous NSHD findings (2,17,22–26). Anthropometric measures, health conditions, and behaviors were assessed at ages 53, 60–64, and 69. Height and BMI (kg/m^2^), derived from nurse-measured height and weight, were considered as continuous measures (23). Four chronic health conditions were ascertained using a series of self-reported questions on knee pain, respiratory symptoms, history of diabetes, and cardiovascular events (2). Individuals reported the frequency they participated in sports, vigorous leisure activities or exercise (never, 1–4 times/month, 5+ times/month) and whether they smoked cigarettes (never, past smoker, current smoker) (24).

Paternal occupational class, reported at age 4 (or ages 11 or 15 if missing at age 4 [*n* = 45]), and own occupational class, reported at age 53 (or ages 43 or 36 if missing at age 53 [*n* = 83]), were based on the Registrar General’s Social Classification (25) and were grouped into three categories: I (professional) and II (intermediate); IIINM (skilled nonmanual) and IIIM (skilled manual); IV (partly skilled manual) and V (unskilled manual) (27). Maternal education was classified into four categories: primary only; primary and further education; secondary only; secondary and further education.

Educational attainment by age 26 was categorized into five groups: degree or higher; A levels or equivalent (typically attained at age 18); O levels or equivalent (typically attained at age 16); clerical course or equivalent; and none. While education is often conceptualized as a measure of socioeconomic position (SEP) (28), here, it is considered separately as a mediator due to the clearly established pathway from childhood cognition to education and adult cognition (17). Adult verbal memory was measured at each age using a 15-item word-learning task that assesses fluid ability (17,22). The total score (max: 45 over three trials) was standardized to the analytical sample such that the mean was 0 and the *SD* was 1. As multilevel models assume that any missing data are missing at random, we also adjusted for binary indicators for mortality (ie, died between ages 53 and 69) and attrition (ie, permanent attrition between ages 53 and 69 not due to death) (26).

### Statistical Analyses

Repeated measures multilevel models (MLMs) were used to examine the associations between childhood cognition and balance time and to assess if this changed with age. MLMs, with fitted random intercepts and slopes, allow for variation both between individuals and within individuals over time (29). The age intercept was set to zero at age 53 for all models. Due to the skewed distribution of balance times, balance was log-transformed and all estimates are presented as percent change in balance time (30). Initial models tested if the association between childhood cognition and balance deviated from linearity and if there were any interactions between childhood cognition and sex or age. This selected model was then adjusted for sex, death, attrition, and anthropometric measures. The following covariates were then added sequentially: chronic health conditions, health behaviors, socioeconomic indicators, education, and verbal memory. Any nonlinear and sex by age interaction terms were included where relevant.

The analytical sample included individuals with a balance time at one or more ages, childhood cognition score and complete covariate data (*n* = 2,380; Supplementary Figure 1). Sensitivity analyses were conducted to test the sex-adjusted association in the maximal available sample and to test all models in the sample with a value of zero imputed where scores were missing due to health reasons (166 additional observations, but only *n* = 49 additional participants) in keeping with previous analyses in NSHD (16).

## Results

Men had higher childhood cognition, educational attainment, occupational class, and balance times at all ages than women, but lower verbal memory (all *p* < .001; Table 1). Men were also less likely to have respiratory symptoms and more likely to have a history of diabetes and cardiovascular events and to have died between ages 53 and 69 (all *p* < .05; Table 1). Those excluded due to missing covariate data (*n* = 405) were similar to the main analytical group (*n* = 2,380), but were more likely to have a lower childhood SEP (*p* < .01), to be a current or ex-smoker (*p* < .05) and to have lower childhood cognition (*p* < .001).

**Table 1.** Characteristics of Analytical Sample (Those With Data on Childhood Cognition, One or More Measure of Balance Ability, Complete Covariates) in NSHD (*n* = 2,380) by Sex

Variable		Men (*n* = 1,185)	Women (*n* = 1,195)
Childhood cognition, mean (*SD*)		0.12 (1.01)	−0.04 (0.95)
Balance time (s), median (IQR)			
Age 53		5 (3–10), *n* = 1,101	4 (3–7), *n* = 1,134
Age 60–64		3.79 (2.53–5.73), *n* = 829	3.27 (2.25–4.83), *n* = 902
Age 69		2.98 (1.98–4.87), *n* = 803	2.91 (1.91–4.31), *n* = 817
Height (m), mean (*SD*)			
Age 53		1.75 (0.06), *n* = 1,126	1.62 (0.06), *n* = 1,170
Age 60–64		1.75 (0.07), *n* = 862	1.62 (0.06), *n* = 935
Age 69		1.74 (0.06), *n* = 830	1.60 (0.06), *n* = 869
BMI (kg/m^2^), mean (*SD*)			
Age 53		27.4 (4.1), *n* = 1,126	27.3(5.2), *n* = 1,167
Age 60–64		27.9 (4.1), *n* = 861	28.0(5.5), *n* = 935
Age 69		28.1 (4.5), *n* = 842	28.2(5.7), *n* = 873
Paternal occupational class, *n* (%)			
I professional/II intermediate		320 (27.0)	308 (25.8)
III skilled (nonmanual or manual)		583 (49.2)	586 (49.0)
IV partly skilled/V unskilled		282 (23.8)	301 (25.2)
Maternal education, *n* (%)			
Secondary and further education		141 (11.9)	143 (12.0)
Secondary only		143 (12.1)	131 (11.0)
Primary and further education		190 (16.0)	164 (13.7)
Primary only		711 (60.0)	757 (63.3)
Own occupational class, *n* (%)			
I professional/II intermediate		620 (52.3)	444 (37.2)
III skilled (non-manual or manual)		440 (37.1)	509 (42.6)
IV partly skilled/V unskilled		125 (10.6)	242 (20.3)
Knee pain, n (%)			
Age 53		169 (15.1)	237 (20.4)
Age 60–64		172 (19.9)	224 (23.8)
Age 69		147 (17.4)	187 (21.4)
Respiratory symptoms, *n* (%)			
Age 53		219 (19.4)	211 (18.0)
Age 60–64		173 (19.8)	174 (18.1)
Age 69		205 (25.2	200 (21.9)
History of angina, stroke or MI, *n* (%)			
Age 53		65 (5.8)	34 (2.9)
Age 60–64		103 (11.7)	46 (4.9)
Age 69		142 (16.3)	87 (9.6)
History of diabetes, *n* (%)			
Age 53		37 (3.1)	26 (2.2)
Age 60–64		92 (9.4)	74 (7.0)
Age 69		128 (13.3)	104 (10.2)
Leisure time physical activity, *n* (%)			
Age 53	None	513 (45.5)	573 (48.9)
	1–4 times/month	222 (19.7)	198 (16.9)
	5+ times/month	393 (34.8)	402 (34.3)
Age 60–64	None	546 (64.5)	574 (62.1)
	1–4 times/month	110 (13.0)	142 (15.4)
	5+ times/month	191 (22.6)	208 (22.5)
Age 69	None	520 (59.4)	578 (60.0)
	1–4 times/month	99 (11.3)	128 (13.3)
	5+ times/month	257 (29.3)	258 (26.8)
Smoking status, *n* (%)^a^			
Age 53	Current	254 (22.5)	259 (22.1)
	Previous smoker	575 (51.0)	527 (44.9)
	Never smoker	299 (26.5)	387 (33.0)
Age 60–64	Current	111 (12.4)	117 (12.1)
	Previous smoker	519 (58.2)	507 (52.3)
	Never smoker	262 (29.4)	346 (35.7)
Age 69	Current	98 (10.4)	91 (9.0)
	Previous smoker	590 (62.3)	573 (56.8)
	Never smoker	259 (27.4)	345 (34.2)
Educational attainment, *n* (%)			
Degree or higher		174 (14.7)	70 (5.9)
GCE A level or Burnham B		343 (29.0)	284 (23.8)
GCE O level or Burnham C		176 (14.9)	308 (25.8)
Sub GCE		72 (6.1)	110 (9.1)
None attempted		420 (35.4)	423 (35.4)
Verbal memory, mean (*SD*)			
Age 53		23.0 (6.2), *n* = 1,126	24.9 (6.2), *n* = 1,164
Age 60–64		23.1 (5.91), *n* = 839	25.2 (6.03), *n* = 914
Age 69		21.2 (5.99), *n* = 821	23.2 (6.02), *n* = 852
Death by age 69, *n* (%)		116 (9.8)	78 (6.5)
Attrition by age 69 (excl. death), *n* (%)		218 (18.4)	217 (18.2)

In men, median balance time decreased from 5 seconds (Q1, Q3: 3, 10; *n* = 1,101) at age 53–3.79 seconds (2.53, 5.73; *n* = 829) at age 60–64 and 2.98 seconds (1.98, 4.87; *n* = 803) at age 69. In women, median balance time decreased from 4 seconds (3, 7; *n* = 1,134) at age 53–3.27 seconds (2.25, 4.83; *n* = 902) at age 60–64 and 2.91 seconds (1.91, 4.31; *n* = 817) at age 69 (Table 1).

**Figure 1.**
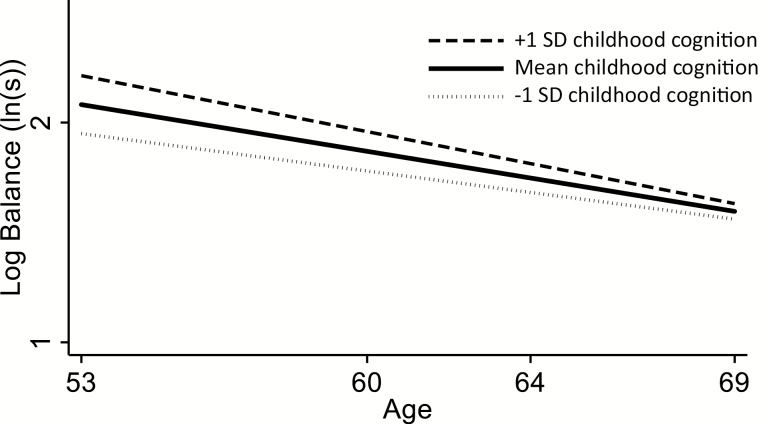
Predicted mean log-transformed balance by age for different levels of childhood cognition from a sex-adjusted multilevel model (*n* = 2,785, obs = 6,379)

Sex-adjusted multilevel models (*n* = 2,380, obs = 4,926), including a childhood cognition by age interaction, demonstrated that a 1 *SD* increase in childhood cognition was associated with a 13% (95% confidence interval [CI]: 10%, 16%) increase in balance time at age 53 (*p* < .001; Table 2, Model 1). The interaction between childhood cognition and age indicated that this association weakened over time (childhood cognition × age interaction term: by −0.6% per *SD* cognition for every year increase in age [95% CI: 0.3%, 0.8%], *p* < .001; Table 2, Model 1; Figure 1). Thus, a 1 *SD* increase in childhood cognition was associated, in sex-adjusted models, with 7% (5%, 9%) and 4% (1%, 7%) increases in balance times at ages 60–64 and 69, respectively.

**Table 2.** Results from Multilevel Models Demonstrating Percent (%) Difference in Mean Balance Time by Childhood Cognition (*n* = 2,380 Individuals, 4,926 Observations)

Model	Percent Difference in Balance Score at Age 53 Per *SD* of Childhood Cognition [intercept]		Childhood Cognition (*SD*) × Age (Year) Interaction	
	Coefficient (%) (95% CI)	*p* Value	Coefficient (%) (95% CI)	*p* Value
1: age^a^, sex^b^	13 (10, 16)	<.001	−0.6 (−0.8, −0.3)	<.001
2: model 1+ death + attrition^c^	12 (10, 15)	<.001	−0.5 (−0.8, −0.3)	<.001
3: model 2 + anthropometric^d^	12 (9, 15)	<.001	−0.6 (−0.8, −0.3)	<.001
4: model 3 + chronic health conditions^e^	12 (9, 14)	<.001	−0.6 (−0.8, −0.3)	<.001
5: model 3 + health behaviors^f^	11 (8, 13)	<.001	−0.5 (−0.8, −0.3)	<.001
6: model 3 + SEP^g^	8 (5, 11)	<.001	−0.6 (−0.8, −0.3)	<.001
7: model 3 + education^h^	6 (3, 10)	<.001	−0.4 (−0.7, −0.04)	.03
8: model 3 + verbal memory^i^	9 (6, 12)	<.001	−0.6 (−0.8, −0.3)	<.001
9: fully adjusted^j^	3 (−1, 7)	.15	−0.3 (−0.7, −0.02)	.04

^a^Age is centered at age 53 = 0 in all models.

^b^Adjusted for age, sex, age × sex (note: age × sex interaction indicates that sex differences in balance ability decreased with age).

^c^Adjusted for model 1 + death, attrition, death × age, death × sex, death × age × sex.

^d^Adjusted for model 2 + height, height^2^, BMI.

^e^Adjusted for model 3 + respiratory symptoms, knee pain, history of diabetes, history of cardiovascular events.

^f^Adjusted for model 3 + smoking history, leisure time physical activity.

^g^Adjusted for model 3 + maternal education, paternal social class, adulthood social class.

^h^Adjusted for model 3 + educational attainment by age 26, age × educational attainment by age 26.

^i^Adjusted for model 3 + verbal memory.

^j^Adjusted for all covariates in Models 1–8.

The associations between higher childhood cognition and better balance times at the intercept (age 53) remained fairly constant with the addition of death and attrition (Table 2, Model 2), anthropometric indicators (Table 2, Model 3), chronic health conditions (Table 2, Model 4) and health behaviors (Table 2, Model 5). The addition of socioeconomic indicators (Table 2, Model 6), educational attainment (Table 2, Model 7) and verbal memory (Table 2, Model 8) partially attenuated the association between childhood cognition and balance, with the lowest intercept estimate of 6% ([3%, 10%], *p* < .001; Model 7) in the education-adjusted model. In the fully adjusted model, the estimates were further attenuated (3% [−1%, 7%] at 53 y/intercept, *p* = .15; Model 9).

When the sex-adjusted model was repeated in the maximal available sample (*n* = 2,785; obs = 6,379), the estimates remained consistent with those in the restricted sample (Figure 1). When scores that were missing due to health reasons were included (imputed with balance time of 0 second), the main findings did not change (Supplementary Table 1).

## Discussion

Higher childhood cognition was associated with better balance performance in midlife, with diminishing associations with increasing age. This association remained robust to adjustment for death, attrition, anthropometric factors, chronic health conditions, and health behaviors. It was largely explained by education, adult verbal memory, and other socioeconomic indicators, suggesting that the association between childhood cognition and later life balance acts largely via cognitive and socioeconomic pathways.

Our findings are consistent with two previous sets of analyses in NSHD and one other Danish study demonstrating an association between higher cognition in early life and better balance performance in midlife (10,11,23). Our study builds on this evidence by demonstrating that although the sex-adjusted association is strongest in midlife (at age 53), it remains at later ages (ages 60–64 and 69). The attenuation in effect size after adjustment for SEP, education, and verbal memory suggests that these factors may mediate the association between childhood cognition and balance.

### Explanations of Findings

The reliance of balance on higher level cognitive processes could extend back past current cognitive ability to the initial formation of neural connections in early life. The cerebellum, an area of the brain heavily involved in balance, undergoes substantial development early in life (7,31) with continual maturation until approximately 15 years of age (32). Childhood may represent a sensitive period necessary for the development of important synapses and neural connectivity involved in successfully balancing. As cognitive ability in childhood is the start of a lifelong cognitive pathway (16–18), it may provide the earliest opportunity for successful interventions.

Our findings suggest that the associations between early life cognition and balance may be mediated by education, adult verbal memory, and SEP. First, balance ability across life is strongly dependent on cognitive processes involved in sensori-motor integration of the nervous system. This is consistent with partial attenuation of the estimates when education and adult verbal memory were added to the model. It is known that cognitive ranking remains fairly consistent across the life course (17,18,33), as such cognition in adulthood may play a role on the pathway between childhood cognition and balance. Education is also likely to play a role on the pathway between childhood and adult verbal memory, as higher cognitive ability in early life correlates with higher educational attainment and subsequent higher fluid ability in adulthood (17).

Second, SEP also attenuated the association when added to the model. Education and adult verbal memory are highly correlated with other indicators of SEP (such as adult occupational class in this study) (25) and as such, these effects are likely to act on the same cognitive-education pathway described above. The association between low SEP and balance impairment could also be mediated by unhealthy behaviors, decreased access to material resources and services to maintain health or increased cumulative life stress (34). However, the addition of health behaviors and chronic health conditions into earlier models had little impact on the main association. Other studies in NSHD and elsewhere have reported associations between lower childhood cognition and poorer physical capability (9,10,22), higher morbidities (33) and premature mortality (35,36) in later life. Thus, in addition to childhood cognition acting as an indicator of neurological function involved in the balance process, this evidence suggests that it is also a predictor of overall health in older age.

We observed that the association between childhood cognition and balance was smaller at older ages. This suggests that there is an advantage of higher childhood cognition on midlife balance but that this advantage gets smaller with age, or equivalently that those with higher cognition in childhood having a steeper decline in balance ability. This advantage in midlife may decrease as age-related impairments in visual, vestibular, or musculoskeletal cues begin to emerge. These age-related impairments may lead to differences in balance ability between older adults that are not observed in younger adults. Individual variation in these more proximal factors, such as sarcopenia and multiple morbidities may thus begin to outweigh balance capability, and its reliance on cognition, developed earlier in life. Further consideration of age-related change in the sensory input systems involved in balance is needed.

### Strengths and Limitations

This study has several important strengths. Firstly, the availability of longitudinal, prospectively ascertained data on cognitive ability and multiple measures of balance performance offered a novel opportunity to investigate this association. As the sample was age homogenous, there was no confounding by age. Cognitive ability and other covariates were prospectively collected, thus limiting recall bias. The use of multilevel models in the analyses increased the statistical power by allowing us to include individuals with any balance data over the three clinical visits from ages 53 to 69 (three balance scores: *n* = 1,329, two balance scores: *n* = 548, one balance score: *n* = 503).

Missing data due to loss to follow-up, death, inability to complete balance assessments due to health reasons and incomplete data on covariates could bias the results as it is known that those who were lost to follow-up tended to have poorer health than those included in the study sample (19,37,38). We did adjust for indicators of death and attrition, examined the sex-adjusted model in the maximal available sample and conducted a sensitivity analysis that included those who were unable to complete the assessment due to health reasons; the results did not change. We modeled the association in various stages to identify factors that may mediate the association between childhood cognition and balance. Consequently, we adjusted for intermediate variables on the causal pathway (i.e., education, adult verbal memory); as such, the impact of adjustment needs to be interpreted with caution.

### Implications

Establishing neural pathways early in life may influence peak balance ability and have long-term advantages in the face of age-related decline in the systems underlying balance ability. Understanding how cognition across all stages of life may impact balance ability and its decline could help inform interventions to combat physical decline (39,40). This could contribute to improving peak balance ability, delaying the onset of balance decline and minimizing rate of decline in mid and later life. Early life intervention studies designed to improve childhood cognitive potential and its long-term consequences (41) should include adult balance ability as an outcome. Ongoing research is currently investigating bidirectional associations between adult cognition and balance ability to better understand the mechanism by which cognition may impact balance.

In conclusion, understanding the mechanisms underlying the positive association between higher childhood cognition and better midlife balance performance may have important implications for falls risk and the maintenance of physical capability.

## Funding

This work was supported by the UK Medical Research Council (Programme codes MC_UU_12019/4, MC_UU_12019/1, and MC_UU_12019/2). J.M.B. also receives support from UCL, the Canadian Centennial Scholarship Fund and the Canadian Institute of Health Research and D.H.J.D. from the Wellcome Trust (WT107467). The MRC National Survey of Health and Development is funded by the UK Medical Research Council. The funders of the study had no role in the study design, data collection, data analysis, data interpretation, writing of the report or the decision to submit the article for publication.

## Conflict of Interest Statement

None declared.

## Supplementary Material

gly275_suppl_Supplementary_Material
