# Supplementary material for: Childhood Cognition and Age-Related Change in Standing Balance Performance From Mid to Later Life: Findings From a British Birth Cohort
Source: J Gerontol A Biol Sci Med Sci. 2018 Dec 8;75(1):155–61. doi: 10.1093/gerona/gly275 (PMC6909897; doi:10.1093/gerona/gly275)
Supplement: gly275_suppl_Supplementary_Material [file gly275_suppl_supplementary_material.docx]

**Age 60-64^i^**

**Age 53^i^**

**Age 69^i^**

Birth

n=5362

Dead, n=469

Living abroad or emigrated, n=580

Refusal, n=668

Unable to contact, n=610

Questionnaire only, n=47

Dead, n=778

Living abroad or emigrated, n=584

Refusal, n=594

Unable to contact, n=1177

Dead, n=995

Living abroad or emigrated, n=583

Refusal, n=961

Unable to contact, n=586

Questionnaire only, n=77

Incapacity protocol, n=11

Clinical assessment

n=2988

Clinical assessment

n=2229

Clinical assessment

n=2149

Valid balance score,

eyes closed

n=2792 (93.4%)

Valid balance score,

eyes closed

n=1983 (92.3%**)**

Valid balance score,

eyes closed

n=2114 (94.8%)

One or more balance scores, eyes closed

**n=3023**

Child cognition score

**n=2785**

**Analytical sample (complete cases)**

**n=2380 (85.5%)**

Missing covariates

n=405 (**14.5%)**

**Supplementary Figure 1.** Flowchart indicating status of all original study members at ages 53, 60-64 and 69 and where the final analytical sample was drawn from

^i^ Cumulative numbers at each subsequent wave; each summing to 5362

**Supplementary Table 1.** Sensitivity analyses including zero imputation for those who were missing data due to health reasons: multilevel models of associations of childhood cognition with balance ability (n= 2429 individuals, 5092 observations)

|  | **Percent difference in balance score at age 53 per SD of childhood cognition [intercept]** | | **Childhood cognition (SD) *age (year) interaction** | |
| --- | --- | --- | --- | --- |
|  | Coefficient (%) (95% CI) | *p-value* | Coefficient (%)  (95% CI) | *p-value* |
| **Model 1:** age ^a^, sex ^b^ | 15 (12, 18) | <0.001 | -0.5 (-0.8, -0.3) | <0.001 |
| **Model 2:** model 1+ death + attrition ^c^ | 14 (11, 17) | <0.001 | -0.5 (-0.7, -0.2) | <0.001 |
| **Model 3:** model 2 + anthropometric ^d^ | 13 (10, 16) | <0.001 | -0.5 (-0.8, -0.3) | <0.001 |
| **Model 4:** model 3 + chronic health conditions ^e^ | 13 (10, 15) | <0.001 | -0.5 (-0.8, -0.3) | <0.001 |
| **Model 5:** model 3 + health behaviours ^f^ | 12 (9, 14) | <0.001 | -0.5 (-0.7, -0.2) | <0.001 |
| **Model 6:** model 3 + SEP ^g^ | 9 (6, 12) | <0.001 | -0.5 (-0.8, -0.3) | <0.001 |
| **Model 7:** model 3 + education ^h^ | 8 (4, 12) | <0.001 | -0.4 (-0.7, -0.04) | 0.03 |
| **Model 8:** model 3 + adult cognition ^i^ | 10 (7, 13) | <0.001 | -0.5 (-0.8, -0.3) | <0.001 |
| **Model 9:** fully adjusted ^j^ | 4 (-0.1, 8) | 0.06 | -0.4 (-0.7, -0.03) | 0.03 |

^a^ age is centered at age 53 = 0 in all models

^b^ Adjusted for age, sex, age*sex (note: age*sex interaction indicates that sex differences in balance ability decreased with age)

^c^ Adjusted for model 1 + death, attrition, death*age, death*sex, death*age*sex

^d^ Adjusted for model 2 + height, height^2^, BMI

^e^ Adjusted for model 3 + respiratory symptoms, knee pain, history of diabetes, history of cardiovascular events

^f^ Adjusted for model 3 + smoking history, leisure time physical activity

^g^ Adjusted for model 3 + maternal education, paternal social class, adulthood social class

^h^ Adjusted for model 3 + educational attainment by age 26, age*educational attainment by age 26

^i^ Adjusted for model 3 + adult verbal memory

^j^ Adjusted for all covariates in models 1-8

**Supplementary Figure 1.** Flowchart indicating status of all original study members at ages 53, 60-64 and 69 and where the final analytical sample was drawn from

^i^ Cumulative numbers at each subsequent wave; each summing to 5362
